# Impact of glucose metabolism on the developing brain

**DOI:** 10.3389/fendo.2022.1047545

**Published:** 2022-12-23

**Authors:** Marta Cacciatore, Eleonora Agata Grasso, Roberta Tripodi, Francesco Chiarelli

**Affiliations:** Department of Pediatrics, University of Chieti, Chieti, Italy

**Keywords:** glucose metabolism, hyperglycemia, hypoglycemia, type 1 diabetes, brain

## Abstract

Glucose is the most important substrate for proper brain functioning and development, with an increased glucose consumption in relation to the need of creating new brain structures and connections. Therefore, alterations in glucose homeostasis will inevitably be associated with changes in the development of the Nervous System. Several studies demonstrated how the alteration of glucose homeostasis - both hyper and hypoglycemia- may interfere with the development of brain structures and cognitivity, including deficits in intelligence quotient, anomalies in learning and memory, as well as differences in the executive functions. Importantly, differences in brain structure and functionality were found after a single episode of diabetic ketoacidosis suggesting the importance of glycemic control and stressing the need of screening programs for type 1 diabetes to protect children from this dramatic condition. The exciting progresses of the neuroimaging techniques such as diffusion tensor imaging, has helped to improve the understanding of the effects, outcomes and mechanisms underlying brain changes following dysglycemia, and will lead to more insights on the physio-pathological mechanisms and related neurological consequences about hyper and hypoglycemia.

## 1 Introduction

The mechanisms and processes of brain maturation are among the most fascinating aspects of human physiology and anatomy. Despite brain remodeling occurs continuously throughout life, the first and most relevant stages of its maturation take place in the first two decades.

The newborn’s brain is about a quarter or a third of the size of the adult’s brain and grows and specializes according to a precise genetic program (1, 2). Because of the interaction with the external environment and the experiences acquired, the dendritic ramifications increase enormously, as well as the synaptic connections that undergo remodeling and “pruning” processes throughout all life (3). The massive structural, volumetric and connectomic changes that occur in the first years of life require adequate energy substrates. Therefore, a correct supply of substrates and a dynamic brain metabolism is fundamental for these changes.

We hereby evaluate the cerebral changes of glucose metabolism in children and examine the impact of hypoglycemia and hyperglycemia on the developing brain.

## 2 Glucose metabolism in the developing brain

The human brain is the most metabolically and energetically expensive tissue within all organs, and glucose is the predominant organic fuel used in all animal species, including humans (4, 5).

The adult human brain consumes approximately 20-25% of the total amount of glucose used by the body, whereas the growing brain consumes an even greater amount (6, 7), with some estimates suggesting that the glucose consumption in the infant brain constitutes more than 40% of the body’s basal metabolic rate (8).

### 2.1 Glucose uptake

While the importance of glucose for proper brain function has been a long-established concept, the exact mechanism by which glucose is able to reach brain tissue is a much more recent discovery. Glucose, as a hydrophilic and polar compound, is unable to spontaneously diffuse across the endothelial membrane. For this reason, the conformation of the blood-brain barrier, with tight junctions between endothelial cells, requires specific transcellular glucose transporters from the blood to the brain. This family of glucose transporters (GLUT) is responsible for the entry of glucose into cells, mediating more than 95% of glucose transport to nervous tissues (9, 10). Several members of this family of transporters have been found in the brain: GLUT 1 to 5 and, more recently, GLUT6 (previously referred to as GLUT9), GLUT8, GLUT10 and GLUTX1 (11). Among these, the most important ones are GLUT 1 and GLUT 3 (12, 13). These transporters exhibit regional heterogeneity in the brain tissue and their expression is regulated at transcriptional, post-transcriptional and post-translational levels by external stimuli, including hypoxia, insulin, hyperglycemia and hypoglycemia, and increased brain glucose demand (5, 9). However, glucose uptake in neurons is independent from the action of insulin, relying on the extracellular concentration of glucose; this exposes the brain to a higher chance of damage compared to other human cells (14).

### 2.2 Brain glucose consumption

Approximately 30% of circulating glucose levels are located in brain extracellular fluid, with about 20-30 minutes of stabilization time during periods of glycemia’s alterations (15, 16). Not all glucose is metabolized immediately, but a great part is stocked in the form of glycogen and held within astrocytes (15). Most of the energy produced from glucose metabolism (70%) is used for neuronal signal transmission functions such as action potential, calcium activities, synaptic transmission, and glutamate cycling; the remaining part is involved in non-signaling activities, like axonal transport, resting potential, and cytoskeleton remodeling. Furthermore, glucose metabolism provides the carbon used for nucleic acids, fatty acids and amino acids synthesis, and produces metabolites that are involved in the regulation of inflammatory and redox reactions (17–20).

Brain glucose consumption can be quantified using the Cerebral Metabolic Rate for Glucose (CMRG), measured by knowing the cerebral blood flow and the arteriovenous glucose difference in the brain, or estimated using fluorodeoxyglucose positron emission tomography (FDG- PET) (5). Because of the invasive aspects of the procedure and the ethical issues in conducting radiodiagnostic investigations in children, there are few studies performed on healthy children: most of them are conducted in young patients with epilepsy, with suspicion of hypoxic-ischemic damage or neonatal hypoglycemia, with autism or when malignancy is suspected.

One of the first studies about the difference in brain metabolism between children and adults was published more than 60 years ago (21). This study showed that the CMRG was significantly higher in children compared to healthy young adults. These findings have been confirmed by a subsequent study on 29 healthy children aged between 5 days and 15 years of age, which investigated for transient and sequelae-free neurological events with FDG-PET (22). The study showed that not only the use of glucose changes with age, but also that, depending on the age of the child, glucose is used differently between the areas of the brain. As also showed in animal studies (23–25), during different stages of development cerebral structures with high CMRG determine the predominant behavioral pattern at the particular evolutionary stage (**Table 1**). Moreover, whole brain CMRG was found to be closely correlated with post-conception age and postnatal age, being lower in the preterm infant compared to children aged 50-60 postconceptional weeks (26).

**Table 1:** Fluorodeoxyglucose – positron emission tomography (FDG-PET) patterns according to age.

Age	FDG-PET pattern	Neurological features
Newborns	Subcortical brain structure	Intrinsic reflexes
3 months	Parietal cortex Primary visual cortex Cerebellar hemisphere	Visual-spatial and visual-motor integration
8 months	Dorsolateral and Frontal-occipital cortex	Greater interaction with the environment
>2 years	Global increased glucose consumption	
3-5 years	Twice the value compared to adults	

In the first two years of life, the CMRG is comparable to the one of a young adult, but with different anatomical patterns depending on the months of life. In term newborns, the most metabolically active areas are the thalamus, the cerebellum, the sensorimotor cortex and the basal ganglia at the expense of the visual cortical regions and the frontal cortex (27–29). In preterm infants, compared to term infants, despite the increased metabolic activity persists in the subcortical regions, the cortical areas are metabolically less active and the FDG-PET images show a finer signal (27). Furthermore, brain development is associated with both significant linear and non-linear changes in regional glucose metabolism in various cortical and subcortical structures from birth to adulthood (30). These changes may also correlate with significant modifications in neurometabolic connections involving the fronto-thalamic, fronto-cerebellar and fronto-hippocampal networks, representing a metabolic correlation between age-dependent effects on sensory, motor, and high-level cognitive functional networks (30).

The neonatal behavior, which is characterized by intrinsic reflexes, is mainly dominated by the activity of the subcortical brain structure, whereas at about 3 months of life afinalistic movements leave space for more coordinated movements that require visual-spatial and visual-motor integration. This is reflected by an increase in the CMRG at the level of the parietal cortex, the primary visual cortex and the cerebellar hemispheres. At about 8 months of life, there is an increase in CMRG at the level of the dorsolateral and frontal occipital cortex, which reflects the greater interaction that the child has with the environment in this period of life. Subsequently, from 8 months to 2 years of life, the absolute CMRG for many structures was similar to that found in young adults. Starting from the second year of life there is an increase in the CMRG which reaches twice the value of the one recorded in adults at around 3-5 years of life, to later progressively decrease between 9 and 15 years (22).

These data were confirmed by more recent studies that extended the cohorts to preterm infants and to a larger group of patients (26–32).

Some authors found that brain metabolism, calculated through FDG-PET acquisitions, was significantly different between males and females aged between 15 and 17 years, with an increase in FDG uptake recorded in females (33). This increase in metabolic activity was consistent with the finding reported in previous volumetric studies, that women mature earlier than men (1, 2).

### 2.3 Aerobic glycolysis

Along with the increased consumption of glucose, some studies showed that in childhood there is also an increased use of oxygen in the brain (34).

The metabolic pathway of oxidative phosphorylation is the main supplier of adenosine-50-triphosphate (ATP), producing up to 36 molecules of ATP per glucose molecule, through a series of phases like glycolysis, the citric acid cycle, and the electron transport chain (35, 36). Traditionally, the oxidative metabolism pathways are preferred, unless perturbation of oxygen supply (such as hypoxia/anoxia) or mitochondria impairment occurs (36). However, recent data suggest that in the brain, glycolysis also happens under sufficient oxygen conditions (35, 36). This phenomenon, called *aerobic glycolysis* (AG), it is not exclusive to brain cells: cancer cells, which are characterized by rapid and uncontrolled proliferation, shift glucose consumption towards AG in order to support the biosynthetic reaction needed for cellular growth (7). Moreover, AG is the primary pathway during proliferation of many fast-growing unicellular organisms, regardless of oxygen availability (35). Thus, it is not surprising that in a developing brain, aerobic metabolism is favored.

During early post-natal and childhood, the AG increases enormously accounting for about a third of the total glucose consumption at about 5 years of life (7). This phenomenon could be one of the main reasons for a greater glucose demand - hence, causing an increase in CMRG; AG seems to be necessary for the development of new nerve structures such as synaptic formations, axonal elongation and myelination (36).

## 3 The contribution of radiodiagnostics

The study of brain glucose metabolism has undergone considerable progress over the years thanks to the use of less invasive techniques that are more easily applicable to the pediatric population.

The first studies on brain glucose metabolism were based on the calculation of the CMRG, by estimating the cerebral blood flow and the difference in glucose measured in the arteries and veins of the brain (5). Subsequently, with the use of the FDG-PET it was possible to estimate glucose metabolism by analyzing the concentration of the radiopharmaceutical at the tissue level and calculating the relative Standardized uptake Volume (SUV) (37, 38). Indeed, SUV values provide an alternative for estimating cerebral glucose uptake by showing a good correlation with CMRG values (39).

Today more innovative and less invasive techniques are able to analyze brain metabolism and they have also been used in the pediatric field. Proton Magnetic Resonance Spectroscopy (1HMRS) is an advanced imaging technique used to detect information on the biochemical composition of the tissues analyzed in a non-invasive way (40, 41). 1HMRS processes the signals from the hydrogen protons to determine the relative concentrations of tissue metabolites including choline, N-acetylaspartate, lipids, glutamine, glutamate and glucose (40, 42). Clinical uses in pediatrics include the diagnosis of brain tumors, neonatal disorders such as hypoxic-ischemic encephalopathy, inherited metabolic diseases, traumatic brain injuries, demyelinating conditions and infectious brain injuries. However, routine implementation of 1HMRS is hampered by the lack of measures of control, acquisition protocols and standardized analysis techniques and the lack of a reference spectrum appropriate for the age of the subject, not yet fully available (40, 43).

An alternative approach is given by the spectroscopic analysis with Nuclear Magnetic Resonance (NMR) after infusion of a non-radioactive substance, the 1- (13)C glucose (44, 45). This compound crosses the blood-brain barrier allowing to map the anatomical distribution and quantify the cerebral concentration of glucose (44). Despite the potential applications in the study of abnormalities of brain glucose consumption, the role of 13C NMR in clinical practice is still a subject of speculation (45).

As for the study of cerebral metabolism, many advances have also been made for the morpho-structural study of the brain. Conventional MRI is useful to assess whole brain measurement and differences between white and gray matter. However, few studies managed to explore the differences during development (14). For example, some authors compared structural MRI findings in children with and without diabetes during a 18 months period, demonstrating a significant reduction in growth of cortical gray matter volume, cortical surface area and white matter volume throughout the cortex and cerebellum. In addition, the authors suggested that fluctuating glucose levels in diabetic patients may also be associated with corresponding fluctuations in brain volume, since they found a negative correlation between change in glycemia levels and change in gray and white matter volumes in these children at the time of the scan across longitudinal time points (46).

Over the last decade, a new type of magnetic resonance imaging called diffusion tensor imaging (DTI) has been developed, which is uniquely suited to study the white matter microstructure (47). DTI measures the magnitude and directionality of water diffusion in tissues distinguishing between isotropic diffusion and anisotropy diffusion (47, 48). Among its applications, this technique has been used to evaluate the difference in brain structure between children with DT1 and age-matched controls, which found a significant change in fractional anisotropy and apparent diffusion coefficient in widespread regions of the brain, which may represent early features of injury to myelinated fibers and/or axon degeneration (49).

## 4 The impact of abnormalities of glucose homeostasis on the developing brain

Childhood and adolescence are the most significant times of neurodevelopmental changes (50). Alterations in glucose metabolism during these periods can have a long-term impact on brain development and cognitive function.

Human data and experimental data on animals suggest that both hypoglycemia and hyperglycemia, depending on age and severity, can alter brain structures and cognitive functions (51–57). In fact, it is proven by many *in vitro* studies that stable glucose levels are fundamental for neuronal functionality and activity (15, 18–20). Periods of abnormal glucose levels impact negatively neuronal activity and survival, as well as situations of rapid glucose fluctuation can cause neuronal injury (15).

### 4.1 Effects of hyperglycemia

As previously mentioned, the uptake of glucose is dependent from its extracellular concentration, exposing neurons to damage when exposed to hyperglycemia (14). Brain cell injury is induced by different mechanism. First, the increase in glucose levels induces an increase in the permeability of the blood brain barrier, which allows the entry of glucose and other substances capable of damaging the central nervous system (58, 59). Glucose can react with intra and extracellular compounds, triggering the production of reactive oxygen species, subsequently leading to oxidative stress. This will later cause a modification of cellular molecules, which will therefore lose their functionality leading to mitochondrial dysfunction and cellular damage (14).

If this event occurs early, the resulting alterations in the brain organization could make the brain more susceptible to future events in chronic conditions such as type 1 diabetes (T1D). Indeed, chronic hyperglycemia can lead to the formation of end products of advanced glycation, increase oxidative stress and even neurodegradation (56, 60, 61).

Similarly to streptozotocin-induced diabetes animal models, which show *in-vivo* degenerative changes of neurons and glia, disarrangement of myelin sheaths and reduced myelin content, glucose variability may damage developing neurons in children (53, 56, 57, 61). In addition, hyperglycemia can induce changes in the composition of brain sphingolipids (ceramides and sphingomyelin) causing membrane rearrangements in some cell populations (62).

#### 4.1.1 The relevance of T1D

The most important pathology determining alterations of glucose homeostasis in the pediatric population is T1D (63). The negative impact of dysglycemia on brain structure and function has been extensively studied in children and adolescents with T1D, leading to the description of common findings of volumetric and structural brain alterations (64) (**Table 2**).

**Table 2:** Neuro-radiological findings in pediatric type 1 diabetes (T1D).

CONVENTIONAL MRI	DTI	SPECTROMETRY	FUNCTIONAL MRI
Total and regional reduction in gray and white matter volumes, proportional to the onset of T1D	Widespread fractional anisotropy reduction and lower axial diffusivity in correlation with glycated hemoglobin values Higher fractional anisotropy in children with lower exposure to hyperglycemia	Lower mean N-acetyl aspartate and higher mean myoinositol and choline, suggesting a neuronal loss or function impairment	Hyperactivation of task-positive regions underlying attentional and executive control, to counteract T1D-associated abnormalities in brain structure and facilitate cognitive and behavioral function

The major neuropsychological sequelae are observed in children with onset of diabetes between the first 5-7 years of life (57, 63–65), with cognitive and structural deficits observed shortly after diagnosis (65, 66). Indeed, the phase preceding the diagnosis of diabetes is characterized by a long period of uncontrolled hyperglycemia and consequent neurotoxicity, which represents a crucial moment in the genesis of the brain damage (58).

##### 4.1.1.1 Radiodiagnostics in T1D

Brain imaging studies have shown that T1D is associated with total and regional reduction in gray matter and white matter volumes (44, 66–71). This reduction is proportional to the time of onset of diabetes, with a more drastic change in brain parenchyma in patients who had an earlier onset of T1D compared to the later-onset T1D group (72). Regional differences may be explained by a higher glucose demand required by certain brain regions, such as the frontal and temporal lobes (65). Indeed, it was frequently recognized a decreased mean gray matter in the frontal precentral and temporal regions, but also in the thalamus and insular cortex (70, 73). A study evaluated 144 T1D children aged between 4 and 10 years and 72 healthy children, through two MRI acquisitions performed 18 months apart: children with diabetes had less cortical gray matter growth than controls, with significant localized differences in the left precuneus extending to the left parietal and posterior-occipital region and the right temporal, frontal and parietal lobes. Similarly to what was found for the gray matter, there were also widespread differences in white matter, with slower growth in T1Ds vs. controls, including the splenium of the corpus callosum, bilateral superior-parietal lobe, bilateral anterior forceps, and inferior-frontal fasciculus, with the main findings identified in the right anterior-frontal lobe (74). A subsequent analysis of the same images also showed that differences in brain substance growth in diabetic patients were most pronounced in the younger tiers among the age range (46).

Volumetric white matter abnormalities in children with T1D have also been correlated with impaired connectivity and cerebral microstructures (75–77). Likewise, some authors demonstrated lower axial diffusivity in children with T1D compared to age-matched controls, and related these differences to a higher average exposure to hyperglycemia, suggesting again a disruption of the physiological development of brain myelination and the importance of achieving an optimal glucose control in these patients (78).

Other authors investigated white matter microstructures abnormalities in T1D patients and healthy controls between 9 and 22 years of age, using DTI (77)*;* they demonstrated that in children with T1D the superior parietal lobule had altered DTI parameters compared with controls correlating this alteration to axonal injury (77, 79). Anomalies of axial diffusivity were also found at the level of the temporal area (75, 76). In addition, a significant correlation emerged between the anomaly of axial diffusivity and the values of glycated hemoglobin recorded at the time of acquisition of the radiodiagnostic images (75).

Relevant data on brain alterations resulting from dysglycemia have recently been brought to light by a study carried out through the acquisition of spectrometric images in children with T1D, a decrease in choline and N-acetylaspartate at the level of the pons was found suggesting a neuronal loss or function impairment in that area, with changes in membrane lipids and/or a decreased membrane turnover (80). A reduction in N-acetylaspartate was reported by human and animal studies and, as a marker of neuronal density, remarking the link between chronic hyperglycemia, demyelination and loss of neurons (53, 73). An increase of myoinositol and choline was also reported in T1D patients, demonstrating an alteration of osmolarity, demyelination and glial hypoxic damage (81).

##### 4.1.1.2 T1D and cognitivity

The impact of dysglycemia on cognition and school performance in children has been the topic of many studies, but it is still a subject of considerable controversies (82). Several studies have found worse outcomes in different cognitive domains in children with early onset diabetes. Ferguson et al. compared MRI brain structures and cognitive ability in a group of young adults with long-duration T1D diagnosed during childhood or adolescence (72). The study reported greater lateral ventricular volumes, more prevalent ventricular atrophy and poorer intellectual and information processing abilities in the early-onset T1D group (72). Similar neurological implications were also reported in children with diabetes and early-onset severe hypoglycemia: these patients were found to have a poorer cognitive performance compared to those with late-onset severe hypoglycemia (83).

Changes on intelligence quotient (73), anomalies in learning and memory (81, 84, 85) and differences in the executive functions (81, 86) have also been reported (**Table 3**). Some authors performed a cognitive study on children with diabetes and on healthy controls, observing cognitive differences in children with diabetes in the areas of intellectual ability and executive functions (66). However, these results were not confirmed in a subsequent re-evaluation performed after 18 months (96). Similarly, equivalent cognitive and behavioral performance were recorded among 93 T1D children and 57 non-diabetic controls. The study analyzed functional MRI of children performing an executive function paradigm, and found an increased activation in executive control regions, as well as reduced suppression in the posterior node of the default mode network. Specifically, worse suppression of the default mode network in children with T1D was associated with hyperactivation of task-positive regions underlying attentional and executive control, suggesting that this activation of executive control networks may transiently counteract T1D-associated abnormalities in brain structure in order to facilitate normative cognitive and behavioral function (63).

**Table 3:** Neurocognitive sequelae resulting from the main alterations of glucose metabolism in children with type 1 diabetes.

Clinical Outcomes	T1D	Early Onset T1D	DKA	Poor Glycemic Control	
				Hypoglycemia	Hyperglycemia
**Global intelligence**	-Lower intelligence (crystallized and fluid) (84) -Lower verbal IQ scores in boys (87)	-Lower performance IQ (88)	- Lower performance IQ in moderate/severe DKA group compared with the none/mild DKA group (89)	-Lower verbal IQ (88) -General intelligence deficit in severe hypoglycemia (52)	-Low general cognitive abilities (90)
**Learning and Memory**	-Poorer working memory (84)	-Poorer sustained and divided attention and poorer new learning (81) - Lower verbal and visual learning and memory ability in early onset vs late onset T1D (84)	Lower rates of accurate memory (91) Poorer delayed memory recall and poorer sustained and divided attention (15)	-Poorer working memory, and non-verbal processing speed (81)	- Poorer working memory (81) -Low receptive language function (90) - Poorer long-delay spatial memory at the time of assessment of T1D (92)
**Executive functions**	- Lower psychomotor efficiency (84) -Lower attention and executive function (84)			- Poorer verbal ability (81) -Verbal fluency/language deficits in severe hypoglycemia (52)	- Slow fine motor speed (90) -Reduced spelling performance (93)
**Neuropsychiatric disorders**	-Increased risk in suicide attempts and in most categories of psychiatric disorders (94) -Increased risk of neurodevelopment disorders (95)				-Higher risk of ADHD, ASD (95)

ADHD, attention deficit hyperactivity disorder; ASD, autism spectrum disorder; DKA, diabetic ketoacidosis; IQ, intelligence quotient; T1D, type 1 diabetes.

Another important aspect to consider in the set of T1D, is the relationship between cognitive deficits and diabetic ketoacidosis (DKA). DKA is the most common acute cause of morbidity and mortality in children with T1D, with neurological consequences that present acutely with the development of cerebral edema. Therefore, several studies questioned the long-term neurological effects of DKA on children’s brain, reporting an impact on brain morphology and functionality, especially in memory function (15, 91, 92). Importantly, a study on a cohort of children with T1D found that, compared with patients without or with mild DKA, patients with moderate/severe DKA have lower cognitive scores and altered brain growth after a single episode of DKA (89).

Several studies have also suggested a link between childhood T1D and increased risk of neurodevelopmental disorders, including attention-deficit/hyperactivity disorder (ADHD), autism spectrum disorders (ASD) and intellectual disability (94, 95, 97). A recent large population-based cohort study showed that individuals with T1D bear a significantly higher risk of developing any neurodevelopmental disorders (Hazard Ratio 1.3), ADHD (Hazard Ratio 1.29) and ASD (Hazard Ratio 1.31) compared with matched reference individuals. This risk also increased with higher mean HbA1C levels, demonstrating that poor glycemic control, assessed using time-varying HbA1C, was an independent risk factor for subsequent neurodevelopmental disorders (95).

Long-term outcomes may also differ by gender. In a study conducted on 64 children between 7 and 16 years old, there was a significant decline in performance by age 7 and in the verbal intelligence quotient between the age of 7 and 16. This was exclusively found in boys with onset of diabetes before 6 years old, but not in those with a later onset and not in diabetic girls (87). Furthermore, diabetic children have been found to read more slowly, to make more time-consuming errors than control in both genders and to have less capacity in the use of spatial information and in language skills in males and females respectively.

However, despite the negative impact of the alteration of glucose homeostasis on the developing brain, to date it is still not possible to fully determine what are the possible cognitive consequences (85). Future studies are needed to clarify the extent and long-term effects of these anomalies.

#### 4.1.2 Neonatal hyperglycemia

Birth causes the cessation of the continuous supply of glucose from the mother; therefore the newborn must use his or her own stored substrates, activating glucose regulation mechanisms in the first minutes of life. In newborns glucose levels decrease at 1-2 hours of age and subsequently increase over the first 18 hours. Glycemia remains stable for the next 48 hours and subsequently increase during the third day of life after birth (98).

However, numerous mechanisms can interfere with this balance, causing hyper or hypoglycemia in the perinatal period. High levels of blood glucose are most commonly reported within the first 3 to 5 days of life, as a consequence of limited insulin secretion capacity, increased counter-regulatory hormones, sepsis, parenteral glucose and medications’ administration (e.g. steroids) (99). Moreover, due to environmental (drugs, parental glucose infusion, sepsis, intrauterine growth restriction) and intrinsic factors (e.g. alteration of hormonal regulation with reduced insulin production and reduced suppression in hepatic glucose production), preterm infants have an increased risk of hyperglycemia (99–102), which is inversely correlated to gestational age (103).

However, hyperglycemia may also occur in term infants, especially those treated with therapeutic hypothermia for hypoxic-ischemic encephalopathy (HIE) and, less commonly, in term infants with neonatal diabetes (104).

Among the main neurologic effects of perinatal hyperglycemia the most frequently encountered short-term complications are white matter image changes and intraventricular hemorrhages (98). In a study of term infants with moderate-to-severe HIE who received therapeutic hypothermia, it was found that infants with hyperglycemia have a higher likelihood of having basal ganglia damage or a global pattern of injury compared to infants with normal blood glucose values, but also a higher frequency of normal MRI compared to the hypoglycemic group (105).

In preterm children early exposure to high glucose levels is associated with white matter reduction on imaging, a greater risk of developing intraventricular hemorrhage and small head circumference (106–108).

Evidence of long-term neurodevelopmental outcomes in term infants is lacking and present conflicting results. In term infants, exposure to hyperglycemia in the first hours of life is correlated with an increased risk of moderate to severe cerebral palsy, poor gross motor outcomes (104, 109) and seizures (110). However, in another study the occurrence of hyperglycemia was not associated with an adverse outcome (111).

Similarly, in preterm babies the relationship between neonatal hyperglycemia and neurodevelopmental outcome remains controversial. Moreover, close monitoring of neonatal hyperglycemia does not improve long-term neurodevelopmental outcomes (112), whereas in other studies neurodevelopment impairment, abnormal behavior development and abnormal executive function have been identified (106, 112).

Another cause of hyperglycemia in neonates – although less common – is neonatal diabetes mellitus (NDM). Differently from classic T1D, which onset is the result of the interplay between a genetic predisposition and the environment, NDM is most often caused by monogenic deficits that involve cellular channels (e.g. ATP-sensitive potassium channel, ABCC8) or alteration in the insulin gene (INS). NDM occurs in approximately 1 in 90,000-160,000 live births (113) and should be considered in infants with insulin dependent hyperglycemia, without an alternative etiology, for longer than seven to ten days (114, 115).

Neurodevelopmental problems are frequently reported in NDM, although it is not easy to tell if they are the consequence of the genetic abnormalities or of the glycemic excursions (116). Global developmental delay and impairment in many areas of functioning are among the neurological symptoms reportedly related to NDM that are caused by the KCNJ11 mutation (116), whereas mutations in IER3IP3 are associated with severe epileptic encephalopathy, microcephalia and seizures (117). Interestingly, treatment with sulfonylurea was found to improve the neurological outcome of patients with NDM, supporting the notion that an early diagnosis of NDM may improve the outcome of these children (118).

In a neuroimaging study conducted on children with persistent NDM, different anomalies from those reported in children with T1D, emerged including multiple punctate with matter hyperintensities on the T2 and FLAIR sequences and hyperintensities in the raphe pontes nucleus (114). However, studies about the neurological outcome of children with T1D compared to the ones with NDM are lacking; thus, it is not known if the differences are linked to the causative genetic mutations or to the early exposure to hyperglycemia.

### 4.2 Effects of hypoglycemia

Hypoglycemia is the form of dysglycemia most readily associated with neuronal insult. Indeed, severe hypoglycemia can cause altered consciousness, progressing to seizures or coma and eventually death. Although there is not a universal defined threshold, progressive cognitive dysfunction seems to occur below a blood glucose level of 3.0 to 3.5 mmol/L (119).

The biochemical mechanisms through which severe hypoglycemia cause neuronal damage are not completely known. It may trigger the synaptic release of excessive glutamate causing intracellular calcium toxicity and excitotoxic cellular damage (120); it may cause the overstimulation in addition of the N-Methyl-D-aspartate receptors, resulting in excitotoxicity first and cell damage later (14). In the set of energy restriction (e.g. fasting, prolonged exercise), the brain cells start using alternative fuels for their metabolism like ketone bodies, which can provide up to 60% of metabolic requirements (119, 121). Some animal studies have demonstrated that brain can both benefit and be injured from the use of these metabolites. Indeed, ketones may reduce infarction and edema in response to hypoxia induced injury when exogenously administered, but it is also documented that in situation of endogenic ketoacidosis they seem to reduce cerebral blood flow by increasing levels of vascular permeability factors and vasoconstrictors as endothelin-1 (15, 91).

Not only extremely high or low levels of glycemia are dangerous, but also rapid fluctuations have a negative impact on cells homeostasis. In studies conducted on neural cell culture models it has been demonstrated that approximately 6 hours fluctuations in glucose levels may affect cellular metabolism, through a decrease in mitochondrial activity and the activation of intrinsic apoptotic pathways (19, 119).

Nevertheless, there are currently conflicting opinions about the role of hypoglycemia.

Some reports identify hypoglycemia as a risk factor for mild cognitive dysfunction and structural anomalies (81, 122, 123), while other studies, also performed on animal models, have not found statistically significant results in this regard (70, 96, 124). MRI studies have described how hypoglycemia may compromise normal hippocampus development, with findings of gliosis and reactive neurogenesis (125).

A further aspect to consider is the impact of the frequency of hypoglycemic episodes. A meta-analysis studied the effect of recurrent severe hypoglycemia on cognitive performance in children with T1D and reported a slight but significant decrease in cognitive performance in T1D children with episodes of severe hypoglycemia compared with those without such episodes (52). In particular, statistically significant differences were found on four cognitive domains: intelligence, learning, memory and verbal fluency (52). Neuroimaging studies support these findings, noticing that severe hypoglycemia preferentially targets neurons in the cerebral cortex, particularly in the medial temporal region, including the hippocampus (involved in memory functions), basal ganglia and brainstem. The neuroimaging studies reported in a meta-analysis also documented a reduction in gray matter volume at the left temporal-occipital junction in diabetic children with one or more severe hypoglycemia episodes (68), and a relatively lesser gray matter density in children with a history of severe hypoglycemia (126), with a high prevalence of damage to the hippocampus and the cornu ammonis (52).

#### 4.2.1 Congenital hyperinsulinism

In childhood, the most common cause of hypoglycemia is represented by Congenital Hyperinsulinism (CH), a heterogeneous congenital disorder characterized by a dysregulation in insulin secretion which results in random hypoglycemia associated with low or normal ketones and no metabolic acidosis (127). CH is frequent in neonatal age (typically within the first week of life), but less common in infants, toddlers, and older children (127, 128). From a physio-pathological point of view, in CH there is a uninhibited insulin secretion due to an altered plasma glucose-insulin feedback regulation, which causes unpredictable and severe hypoglycemia, but also represses lipolysis, leading to a reduction of ketones’ formation, the alternative brain fuels to maintain neuronal function (128).

CH usually presents with persistent and unpredictable episodes of hypoketotic hypoglycemia, which are detrimental to the vulnerable neonatal brain and are associated with adverse neurodevelopment (128). A study assessed that a third to half of children with CH present with adverse neurodevelopment regardless of the type of CH (focal/diffuse or transient/permanent) The consequences of perinatal hypoglycemia can vary from MRI-detectable extensive bilateral areas of cystic encephalomalacia in the occipital-parietal area and posterior temporal lobes, to more subtle effects like mild motor delay, definable only by formal cognitive assessment (128). Moreover, brain insult topography is related to the age of hypoglycemic manifestations (129, 130) as demonstrated by the increased risk of pyramidal tract damage in newborns that present hypoglycemia in the first day of life (131).

Not all children have severe and persistent forms of the disease (128), but it is important to early identify them because early-life hypoglycemia has a negative impact on cognitive function of these patients, especially for the ones with an early onset. Indeed, neurodevelopmental abnormalities (e.g. motor and speech impairment) due to delayed diagnosis and suboptimal management are reported in 25%–50% of CH cases (129, 132).

#### 4.2.2 Neonatal hypoglycemia

Hypoglycemia is one of the most common disorders in neonates, occurring in as many as 19% of infants overall (133, 134).

Numerous risk factors, both maternal and fetal, have been identified for neonatal hypoglycemia (NH). The causes of NH can be briefly divided into: a) increased glucose utilization in sick or stressed infants (e.g. neonates with perinatal hypoxia-ischemia or congenital heart diseases); b) hyperinsulinemia due to maternal gestational diabetes, obesity or due to birth asphyxia, placental insufficiency or post maturity; c) inadequate substrate stores in preterm or Intra Uterine Growth Restriction or Small For Gestational Age infants; d) iatrogenic causes, such as hypothermia, malposition of umbilical catheters; e) hormonal deficiencies in infants with hypopituitarism, hypothyroidism, adrenal insufficiency; f) congenital disorders, like Beckwith-Wiedmann syndrome, Down syndrome, Turner syndrome; g) defects of glucose transporter (98). However, most causes of NH are multifactorial (134).

In conditions of hypoglycemia, the newborns’ brain can use alternative energy substrates such as ketone bodies, amino acids and lactate; it also increases cerebral flow, improving the transport of substrates for glycogenolysis, and reduces neuronal activity (135, 136). Anyway, these alternative fuel sources may not be available in preterm or Small For Gestational Ages infants, or may be unable to be used due to perinatal factors such as hypoxia, seizures or pathologic jaundice (137). Therefore, the energy failure caused by consuming or not using alternative substrates may result in cerebral injury (136).

Long term sequelae can occur within a wide range of low serum glucose values, and even transient moderate hypoglycemia may result in neurological damage (138). Cerebral injury caused by NH includes cortical neuronal injury, ischemic stroke, parenchymal hemorrhage and withe matter injury (134, 139–141). Since the first neuroimaging studies, a disproportionate involvement of the occipital and parietal lobes emerged (142). This involvement was then confirmed by subsequent studies which also identified abnormal signals in the deep gray matter structures of the thalamus and basal ganglia (26, 143) and diffusion restriction in the parietooccipital areas, underlying white matter, and corpus callosum (144).

The reasons why the occipital cortex is so sensitive to hypoglycemia in newborn period are not clear. Animal studies have shown a marked increase in synaptic contact in this region during the first weeks of postnatal life. Therefore, the occipital lobe could be particularly prone to damage due to due to glucose availability during this period of continuous remodeling (141). A meta-analysis involving 1657 infants found that in early childhood (2-5 years), exposure to neonatal hypoglycemia was not associated with neuro developmental impairment but was associated with visual-motor impairment and executive dysfunction (109).

Major long-term effects seem to appear at a later age (145). In fact, in mid-childhood (6-11 years), neonatal hypoglycemia was associated with neuro-developmental impairment and low literacy and numeracy (145). Another study compared the long-term outcomes of preterm infants with a history of hypoglycemia with those of normoglycemic controls at 3 to 18 years of age, concluding that no significant differences were observed in cognitive or academic skills between the control and affected groups at any age (146).

## 5 Conclusions

Glucose is the most important substrate for proper brain functioning and development. Its metabolism changes over the years according to a pattern related to the acquisition of more precise ability to interact with the environment. The increased glucose metabolism demonstrated in childhood is related to the need of creating new brain structures and connections; therefore, alterations in glucose homeostasis will inevitably be associated with changes in the development of the Nervous System.

Several studies have highlighted how the alteration of glucose homeostasis leads to a reduction in gray and white matter volume and to brain metabolism alterations. Although the mechanism of brain damage caused by dysglycemic conditions is not yet completely known, structural abnormalities have been predominantly associated with hyperglycemic conditions. This is particularly interesting in relation to the therapeutic habit of preferring higher blood sugar levels in children with T1D, rather than risk of incurring unwanted hypoglycemia.

Our knowledge on the impact and outcomes of dysglycemia on the infant brain are still partial and are almost all derived from studies performed on children diagnosed with T1D, a disease characterized also by insulin alteration and episodes of hyperketonemia. Therefore, it is possible that some of the anomalies found are due to the much more complex pathological picture.

Recent studies about the impact of dysglicemia on cognitive outcomes found that children with T1D had a worse outcome in different cognitive domains, including intelligence quotient, abnormalities in learning and memory and differences in executive functions. Moreover, since differences in brain structure and functionality were also found after a single episode of DKA, one might speculate about the importance of performing screening programs for T1D in order to protect children from the neurological consequences caused by such glycemic alterations.

Data available in newborns are discordant as well, and the study of this population is made even more difficult by the heterogenicity of the comorbidities analyzed in the different trials. However, both hypo and hyperglycemia may cause neurologic injury, which is expressed by a wide range of structural and cognitive abnormalities. Therefore, in high-risk infants it is important to promote strategies to maintain euglycemia and to continuously monitor glucose levels in order to reduce the severity and duration of episodes of dysglycemias.

Further research is necessary in order to improve the understanding of the effects, outcomes and mechanisms underlying brain changes following dysglycemia. New imaging methods, increasingly precise and less invasive, will also improve knowledge in this important field of pediatric research and clinical care.

## Author contributions

Conceptualization, MC and FC. Writing—original draft preparation, MC, EG and RT. writing—review and editing, EG; supervision FC. All authors contributed to the article and approved the submitted version.
